# Frontal synaptic plasticity: A new key to homeostatic sleep regulation

**DOI:** 10.4103/NRR.NRR-D-25-00231

**Published:** 2025-06-19

**Authors:** Yusuke Iino, Shoi Shi

**Affiliations:** International Institute for Integrative Sleep Medicine (WPI-IIIS), University of Tsukuba, Tsukuba, Ibaraki, Japan

Sleep is a fundamental biological process essential for maintaining brain function, cognitive performance, and overall health. Despite over a century of research, the mechanisms underlying sleep homeostasis—the process by which the need for sleep accumulates during wakefulness and dissipates during sleep—remain incompletely understood. This article explores the latest advancements in sleep research, focusing on the role of synaptic plasticity in sleep homeostasis, as illuminated by Sawada et al. (2024).

**The two-process model of sleep regulation:** The foundational framework for understanding sleep regulation is the two-process model, which posits that sleep is governed by the interaction of two processes: sleep homeostasis (Process S) and circadian rhythms (Process C). Process S represents the homeostatic drive for sleep, which increases during wakefulness and decreases during sleep. Process C, furthermore, is controlled by the circadian clock, which regulates the timing of sleep and wakefulness across the 24-hour day (Borbély et al., 2016).

While the two-process model has provided a fundamental framework for understanding sleep regulation, the biological mechanisms underlying Process S—particularly the accumulation and dissipation of sleep pressure—remain elusive. One of the most well-established markers of sleep pressure is electroencephalographic (EEG) delta power, which refers to the low-frequency (1–4 Hz) activity observed during non-rapid eye movement (NREM) sleep. Delta power increases with prolonged wakefulness and decreases as sleep progresses, reflecting the homeostatic regulation of sleep needs.

**Synaptic plasticity and sleep homeostasis:** A hypothesis regarding the biological basis of sleep pressure is that synaptic plasticity—the ability of synapses to change the strength of connections between neurons over time—plays a central role in sleep homeostasis. The synaptic homeostasis hypothesis proposes that wakefulness is associated with synaptic strengthening, or long-term potentiation (LTP), which increases neuronal synchrony and enhances delta power during NREM sleep (Frank, 2013). Conversely, sleep is thought to facilitate synaptic downscaling or long-term depression, which reduces synaptic strength and resets the brain for the next period of wakefulness.

While thalamocortical synapses might show dynamics that contrast with synaptic homeostasis hypothesis (Cary and Turrigiano, 2021), studies have shown that cortical dendritic spine size increases following prolonged wakefulness and undergoes downscaling during sleep, in parallel with reductions in delta power (de Vivo et al., 2017; Suppermpool et al., 2024). However, until recently, there was no direct causal evidence linking synaptic potentiation to increased sleep pressure and delta power.

**Figure 1:**
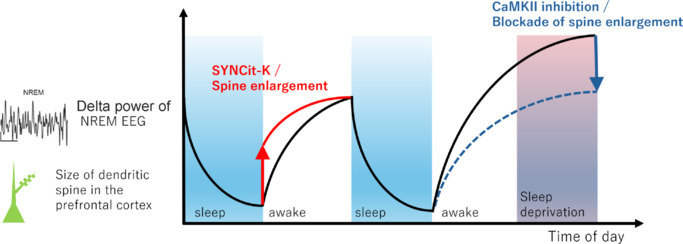
Dendritic spines in the prefrontal cortex regulate sleep pressure. When spine enlargement in the prefrontal cortex was induced using a novel chemogenetic tool called SYNCit-K, the delta power of the NREM sleep EEG increased. Conversely, blocking CaMKII—a key regulator of spine enlargement and long-term potentiation—prevented sleep deprivation from elevating delta power in the NREM sleep EEG. CaMKII: Calcium/calmodulin-dependent protein kinase II; EEG: electroencephalography; NREM: non-rapid eye movement; SYNCit-K: synapse-targeted chemically induced translocation of Kalirin-7.

**The role of the prefrontal cortex in sleep regulation:** A recent study by Sawada et al. (2024) has provided causal evidence that synaptic potentiation in the prefrontal cortex (PFC) directly increases both the amount and depth of NREM sleep, as well as EEG delta power (**Figure 1**). The PFC, a brain region involved in higher-order cognitive functions such as decision-making and working memory, has long been implicated in the generation of slow-wave activity during sleep and sleep homeostasis. The findings from a study by Sawada et al. (2024) reveal that synaptic strength in the PFC causally regulates sleep pressure by using a novel synaptic chemogenetic tool called SYNCit-K (synapse-targeted chemically induced translocation of Kalirin-7), with homeostatic downscaling occurring during NREM sleep to reset synaptic load and reduce delta power.

**Synaptic plasticity generates up-down states and delta waves:** EEG recordings indicate that NREM sleep is characterized by prominent delta waves, which emerge from synchronized transitions between depolarized (up) and hyperpolarized (down) states of cortical neurons (Vyazovskiy and Harris, 2013). During wakefulness, neural activity is desynchronized, producing high-frequency, low-amplitude EEG patterns. These shifts in network activity are governed by changes in synaptic strength, particularly in excitatory cortical circuits.

To investigate how synaptic strength influences sleep pressure and delta waves, Sawada et al. (2024) developed a computational excitatory-inhibitory network model. They found that when excitatory synaptic strength is moderate, the interplay between excitatory and inhibitory balance keeps the system stable, thereby maintaining up-states. In contrast, stronger excitatory coupling overwhelms this balance, destabilizing the network and causing periodic transitions between up and down-states. This destabilization drives large-amplitude, low-frequency population activity, mirroring the delta waves observed during NREM sleep.

To validate these predictions, the researchers pharmacologically enhanced synaptic transmission in cultured cortical neurons using S18986, a positive allosteric modulator of α-amino-3-hydroxy-5-methyl-4-isoxazolepropionic acid receptors (Heckman et al., 2018). This manipulation increased the occurrence of synchronized up-down states and elevated delta power in local field potential recordings, directly supporting the idea that synaptic strengthening amplifies slow-wave activity in cortical networks.

**A chemogenetic tool for synaptic potentiation – SYNCit-K:** A key innovation from Sawada et al (2024). is the development of SYNCit-K, a chemogenetic tool that enables precise and reversible manipulation of synaptic strength. Kalirin-7 is a guanine nucleotide exchange factor involved in dendritic spine remodeling and synaptic potentiation. SYNCit-K employs rapamycin-dependent heterodimerization to direct the guanine nucleotide exchange factor domain of Kalirin-7 to postsynaptic spines, selectively inducing structural LTP.

In both cultured cortical neurons and *in vivo* experiments, the administration of a rapamycin analog triggered Kalirin-7 translocation to dendritic spines, leading to spine enlargement and enhanced excitatory postsynaptic currents. Compared to optogenetic or electrical stimulation-based approaches, SYNCit-K offers a non-invasive means to induce LTP in a temporally and spatially controlled manner.

**Frontal cortex synaptic strength regulates sleep amount and depth:** Using SYNCit-K, Sawada et al. (2024) tested whether targeted synaptic potentiation in the PFC influences sleep. They expressed SYNCit-K in the excitatory neurons of the dorsomedial PFC and administered a rapamycin analog before sleep recordings. Their results showed a significant increase in NREM sleep duration and EEG delta power.

Critically, the same manipulation in the primary visual cortex (V1) did not alter sleep, nor did strengthening synapses in the inhibitory neurons of the PFC. These findings demonstrate that synaptic strength in the excitatory neurons of PFC plays a causal role in regulating sleep need and delta oscillations, aligning with previous evidence suggesting that the frontal cortex is a major source of EEG slow waves (Vyazovskiy and Harris, 2013).

Further supporting the role of synaptic plasticity in sleep homeostasis, inhibition of calcium/calmodulin-dependent protein kinase II (CaMKII), a key regulator of LTP (Lisman et al., 2012), in the PFC suppressed the increase in delta power following sleep deprivation. This suggests that CaMKII-dependent synaptic potentiation is necessary for the accumulation of sleep pressure and its subsequent expression as heightened delta activity during recovery sleep. Given that CaMKII plays a central role in excitatory synaptic strengthening, this result aligns with the hypothesis that wake-dependent LTP mechanisms drive sleep homeostasis by modulating cortical excitability.

**Sleep-dependent synaptic downscaling resets sleep need:** Although SYNCit-K-induced synaptic potentiation persisted for over 24 hours in V1, the associated increase in sleep and delta power lasted only a few hours. To explore this discrepancy, Sawada et al. (2024) monitored dendritic spine size in PFC neurons before and after sleep and found that spines shrank back to baseline levels following NREM sleep.

This observation suggests that while LTP in certain brain regions encodes sleep needs, sleep itself facilitates synaptic downscaling, reducing delta power and restoring baseline synaptic strength. Supporting this notion, pharmacological inhibition of long-term depression prolonged NREM sleep, reinforcing the idea that sleep serves a homeostatic function in resetting synaptic load (Garde et al., 2020).

**Concluding remarks:** The study by Sawada et al. (2024) provides causal evidence that synaptic strength in the PFC regulates sleep homeostasis, linking cortical LTP to increased NREM sleep and delta power. Their findings support a model in which sleep need accumulates through wake-dependent synaptic potentiation and is subsequently dissipated via sleep-dependent synaptic downscaling—although the precise mechanisms underlying both processes remain unclear.

While Sawada et al. (2024) provide causal evidence linking PFC synaptic strength to sleep homeostasis, it also opens up exciting avenues for further exploration. For instance, the modest effect mediated by SYNCit-K encourages future research into cortico-cortical selective manipulations, which may enhance the impact on sleep regulation, given the stability of thalamocortical synapses across the sleep–wake cycle. Moreover, although local synaptic potentiation plays a central role in sleep dynamics, exploring alternative mechanisms—such as the influence of glutamatergic neuron firing in the basal forebrain and the PFC-to-basal forebrain projection—could offer a more comprehensive understanding of sleep regulation (Peng et al., 2020). Finally, observations from Aton et al. (2009), which demonstrated synaptic potentiation in the V1 following sleep after monocular deprivation, hint at regional variations in sleep-dependent synaptic changes. Future studies should investigate how these processes interact with global neuromodulatory systems and cognitive functions such as memory consolidation, ultimately paving the way for innovative treatments for sleep disorders and neuropsychiatric conditions.

**Implications for sleep disorders and neuropsychiatric conditions:** The findings of Sawada et al. (2024) have implications for understanding and treating sleep disorders and neuropsychiatric conditions. For example, insomnia, a common sleep disorder characterized by difficulty falling or staying asleep, may be linked to dysregulation of synaptic plasticity in the PFC. Similarly, conditions such as depression and anxiety, which are often associated with disrupted sleep patterns, may involve abnormalities in synaptic homeostasis.

Future research could explore whether interventions that modulate synaptic plasticity, such as pharmacological agents or non-invasive brain stimulation techniques, could be used to treat these conditions. Additionally, understanding the role of synaptic plasticity in sleep regulation may lead to the development of new biomarkers for sleep disorders, allowing for more accurate diagnosis and personalized treatment approaches.

**The broader context – sleep and cognitive function:** The relationship between sleep and cognitive function is a topic of growing interest in neuroscience. Sleep is known to play a critical role in memory consolidation, learning, and emotional regulation. The findings of Sawada et al. (2024) suggest that synaptic plasticity may be a key mechanism underlying these processes.

For example, the synaptic homeostasis hypothesis posits that sleep-dependent synaptic downscaling is essential for maintaining the efficiency and stability of neural networks. By resetting synaptic strength, sleep may help to optimize brain function, allowing for more effective learning and memory formation during wakefulness.

Future research could explore how synaptic plasticity during sleep interacts with other cognitive processes, such as attention, decision-making, and problem-solving. Understanding these interactions could provide new insights into the role of sleep in cognitive health and inform strategies for enhancing cognitive performance.

**Conclusion:** The study by Sawada et al. (2024) represents a significant advancement in our understanding of the mechanisms underlying sleep homeostasis. By demonstrating that synaptic strength in the PFC plays a causal role in regulating sleep needs and delta oscillations, the study provides compelling evidence for the synaptic homeostasis hypothesis.

The development of SYNCit-K, a novel chemogenetic tool for manipulating synaptic strength, opens new avenues for research into the role of synaptic plasticity in sleep and cognitive function. Future studies could build on these findings to explore the broader implications of synaptic homeostasis for brain health and disease.

As we continue to unravel the mysteries of sleep, the insights gained from studies like this one will be crucial for developing new approaches to treating sleep disorders and enhancing cognitive function. Sleep, once considered a passive state, is now recognized as an active and dynamic process that plays a vital role in maintaining brain health and overall well-being.

*This work was supported by Japan Society for the Promotion of Science (JSPS) Grants-in-Aid for Scientific Research (KAKENHI) (20H05894, 20H05903, 21K15136, 22K21351, 23H02518A, 23H02663, and 23K18147 to SS), JST-CREST (JPMJCR24T4 to SS), the World Premier International Research Center Initiative (WPI) from the Ministry of Education, Culture, Sports, Science and Technology (MEXT) to SS (WPI-IIIS), the Top Runners in Strategy of Transborder Advanced Researches (TRiSTAR) by the MEXT to SS, and Japan Agency for Medical Research and Development (AMED) (JP21zf0127005 to SS), Cell Science Research Foundation Grant to YI, 38*^*th*^ *Brain Science Foundation Research Grant to YI, Research Grant on Biogenic Amines and Neurological Diseases (Sumitomo pharma) to YI.*
